# An avian dominance hierarchy at a supplemental water source in the Patagonian steppe

**DOI:** 10.1371/journal.pone.0244299

**Published:** 2020-12-31

**Authors:** Sophie Rabinowicz, Natalia García, Tristan Herwood, Amanda Lazar, Benjamin Hein, Eliot Miller, Leonardo Campagna

**Affiliations:** 1 College of Agriculture and Life Sciences, Cornell University, Ithaca, New York, United States of America; 2 Fuller Evolutionary Biology Program, Cornell Lab of Ornithology, Ithaca, New York, United States of America; 3 División Ornitología, Museo Argentino de Ciencias Naturales, Bernardino Rivadavia, Consejo Nacional de Investigaciones Científicas y Técnicas (CONICET), Buenos Aires, Argentina; 4 College of Arts and Sciences, Cornell University, Ithaca, New York, United States of America; 5 Macaulay Library, Cornell Lab of Ornithology, Ithaca, New York, United States of America; 6 Department of Ecology and Evolutionary Biology, Cornell University, Ithaca, New York, United States of America; MARE – Marine and Environmental Sciences Centre, PORTUGAL

## Abstract

Birds often compete and engage in interspecific agonistic interactions for access to resources such as food and breeding territories. Based on the observed outcomes from such interactions (i.e., patterns of displacements) dominance hierarchies can be established. Knowing which species can outcompete others for essential resources allows researchers to make predictions about the broader ecological impacts of interspecific interactions. We constructed an interspecific dominance hierarchy of twelve avian species which visited an artificial water source in an arid region of coastal Patagonia, Argentina. Displacements were categorized into four types, based on the behaviors involved in the interaction, and we tested if they could predict the difference in dominance between the interacting species (the difference between calculated dominance coefficients for the two focal species). Indirect displacements, involving only the arrival of the dominant species to the water source without direct aggression toward the subordinate bird, occurred more frequently between species with a large difference in dominance. The most dominant bird observed was the kelp gull (*Larus dominicanus*), which, due to an increasing population and expanding range, in part due to food supplementation from fisheries waste, is likely to outcompete terrestrial and marine avian species for other scarce resources.

## Introduction

Resource scarcity leads to both intra- and interspecific conflict as individuals compete with each other for access by using aggressive behaviors to gain and maintain control of these resources [1, 2]. When there is a consistent pattern of aggressive behavior between individuals or different species, a dominance hierarchy that determines resource access is formed [3–6]. These hierarchies reduce the frequency of aggressive interactions and the possibility of injury or death to individuals as a result [6]. Among birds, interspecific hierarchies are mainly organized based on body size [7, 8], while individual positions in intraspecific hierarchies are affected by age and sex [9]. Subordinate individuals and species in the dominance hierarchy have less access to resources or are forced to use lower-quality ones, ultimately reducing their fitness [10, 11].

Food is the limiting resource used most often to study interspecific agonistic interactions and the associated dominance hierarchies [5, 12]. Among avian species, it is unclear how other essential resources such as water affect interspecies dominance. In arid regions, water scarcity is a particularly important consideration for small terrestrial birds because they are more susceptible to water imbalance than large ones, especially when they are under heat stress [13]. All birds have adaptations that limit their water requirements [14], such as the use of water produced by their oxidative metabolism and the excretion of uric acid instead of urea, and some species, particularly large marine and desert birds, also possess unique adaptations to cope with water limitations [14]. For small terrestrial birds, constant access to freshwater is a major requirement for their survival [15], and therefore we expect interspecific competition for access to this resource.

Despite the importance of water, avian dominance hierarchies have mainly been evaluated using food sources such as bird feeders because they are common in backyards around the world, and agonistic displays, often displacements, are easily observed [5, 10, 12, 16, 17]. In addition, large-scale interspecific dominance hierarchy projects have not yet focused on distinguishing between more subtle variations of displacement behaviors that may be the consequence of the relative position of interacting species in a dominance hierarchy [5, 12]. In the present work, we generated a dominance hierarchy through observation of agonistic bird behavior at an artificial water source in Argentinean Patagonia, and investigate if this hierarchy can be predicted by body size. The dry climate and water scarcity in this region allowed us to study if dominance hierarchies form around freshwater sources, where birds of different species congregate to bathe and drink. Our study takes a nuanced approach by piloting a new methodology that takes variations in the displacement behaviors into account in order to demonstrate that different types of displacements can act as indicators of the relative position of interacting species in the dominance hierarchy This work also shows that interspecific dominance hierarchies can be created at a water source in an arid region.

## Materials and methods

### Data collection

We collected data by recording species interactions at an open 3-meter-wide water tank that was easily accessible to birds and other animals. The tank was located at a sheep ranch in Patagonia, just outside of Bahía Bustamante in the Chubut Province of Argentina (45°07'33.9"S 66°32'14.1"W) and is a source of drinking water for livestock and wildlife. Access to the study site was provided by the owners of the Bahía Bustamante property. This study was observational and did not require the handling of wild animals.

This water tank is the only supplemental water source within several kilometers in this portion of the property, making this tank the main source of freshwater for surrounding wildlife. This region receives very little rainfall throughout the year, averaging 14 mm per month in the summer [18]. All the interactions were recorded in January 2020 by a single observer (SR) located about 15 meters away from the tank and after waiting 5 minutes for the birds to become habituated. All displacements were recorded over 35 10-minute observation sessions with 5-minute breaks between consecutive sessions. All observations were made during 4 days in January 2020. During these days, observations were opportunistic and occurred mainly in the morning and the afternoons, avoiding the hotter period of the day between noon and 2 PM when bird activity was lowest. On average 4.7 displacements occurred during each interval (SD = 2.8). At least one displacement was observed during all but three of the observation intervals. Individuals that arrived at the water tank but left without displacing any individual or being displaced were not recorded. When a single individual displaced multiple individuals in one interaction, all displaced birds were recorded separately. Although we sometimes observed a few individuals of the same species which visited the water tank at the same time, we never observed large flocks which would not have allowed us to make detailed observations of individual behaviors. We did not observe intra- or interspecific chases. In total, 165 displacements were observed, 131 of which were interspecific (S1 Table).

### Displacement classification

The observed displacements were split into four categories, defined as follows:

#### Direct displacement

A dominant individual replaces the position of a subordinate individual, causing it to leave the area.

#### Indirect displacement

A dominant individual’s arrival at or near the tank causes a subordinate individual on the tank to leave the area without physical interaction between the two birds. Although we cannot rule out the possibility that some of these recorded interactions are only coincidental arrivals and departures and not displacement interactions, we believe this is unlikely and are confident that all recorded displacements of this type are the result of indirect aggressive behavior. In most cases, recorded indirect displacements were the result of the departure of every individual at the water tank in response to the arrival of highly dominant birds. Furthermore, for the three most dominant species, we never observed subordinate species remain on the tank during the arrival of dominant species. This type of displacement resembles a common behavior observed in predator-prey interactions, in which a prey animal perceives a predator as a threat and leaves the area to avoid predation [19, 20].

#### Partial displacement

A dominant individual directly displaces a subordinate individual, which changes its location on the tank but does not leave the area.

#### Rejected displacement

An individual attempts to directly displace another individual but is rejected through aggressive behavior. In this case, the subordinate individual is the aggressor that fails to displace the dominant individual, who remains at its original position. Therefore, the outcome is the same as a direct displacement between a dominant and a subordinate individual yet differs in that the latter initiates the interaction.

### Dominance measurement

All recorded interspecific displacement interactions were used to generate species dominance coefficients using the Bradley-Terry model [21] as modified by Miller et al. [5] and implemented in the R [22] package networkTricks [23]. This was chosen over other models because it offers more reliable rankings for loosely connected species [5]. The Bradley-Terry model works by using recorded wins and losses from agonistic interactions and assigning each species a dominance coefficient that predicts the outcome of an agonistic interaction for each species. A high coefficient indicates that a species is more likely to win against a greater number of species in agonistic interactions and is therefore higher up in the dominance hierarchy. Lower coefficients indicate that a species is more likely to lose against a higher number of species in agonistic interactions and is thus ranked lower in the hierarchy. We obtained the same results irrespective of the inclusion or exclusion of intra-specific interactions. We checked if there were intransitivities (rock-paper-scissors relationships; [5, 24]) in the hierarchy using the is.dag() function from the igraph package in R. We displayed the dominance hierarchy visually following the attribute-ordered network method described by Hobson et al. [25]. Briefly, species are ordered by their dominance rank from top to bottom and interactions are shown with lines connecting the species. Blue lines show instances where a dominant species displaced one of a lower rank, while the opposite is shown by red lines.

### Statistical analyses

The recorded 165 interactions involved 12 species, although the numbers of observed interactions varied widely per species. We analyzed the sensitivity of the model to sample sizes by subsampling the data and recalculating the dominance coefficients. To determine whether the dominance coefficients were based on a sufficiently large sample size, we subsampled the data 1000 times and calculated the dominance coefficients in every iteration. Finally, we ranked each species according to their coefficients (from most to least dominant) and we determined the average rank across the 1000 iterations for every species. We also calculated the proportion of the 1000 randomization trials in which each species was present. This procedure was carried out in R, subsampling from 20 to 160 observations in increments of 20 (1000 iterations for each subsample size).

We used Phylogenetic General Least Square (PGLS) regressions to test the relationship between species rank (represented by the dominance coefficients) and body mass as a proxy for body size. We obtained the average mass for each species from Dunning Jr. [26] and the maximum clade credibility tree used in the analysis from Jetz et al. [27], and subsequently pruned it to only include the species in our study using the ape package for R [28]. We then used the package phylolm [29] to run the PGLS regressions under Brownian Motion and Ornstein-Uhlenbeck models of evolution, and kept the best model according to its AIC value, which met the assumptions of normality of the residuals and a lack of correlation between the residuals and the fitted values. Although it is preferable to adjust PGLS models and simultaneously estimate Pagel’s lambda when sample sizes are large [30], the estimation of this parameter is very sensitive to the number of species. We therefore chose not to estimate Pagel’s lambda with our relatively small dataset [31].

We also assessed the relationship between the difference in dominance coefficient between the two species that were involved in a displacement interaction and the displacement type using ANOVA. The difference between the dominance coefficients was calculated for all 131 recorded interspecific displacements and compared to the occurrence of each of the four displacement types. Our data did not meet the assumptions of normality in the response variable or homogeneity of variance. Moreover, because we did not band individual birds there is potential for pseudoreplication as it is possible that some of the same individuals were involved in many different interactions over the sampling period. However, we believe that this is unlikely to have a strong effect in biasing our sampling as most of these species are very common and have large populations in the area. We also obtained the same results using Welch’s ANOVA (which does not assume homogeneity of variance) or a Kruskal-Wallis test (which does not assume normality). Overall, although our data did not meet all the assumptions of the tests that we conducted to find differences in relative dominance coefficients between interaction types, we believe our result is robust, based on a large sample size, and shows a clear relationship.

## Results

**Fig 1:**
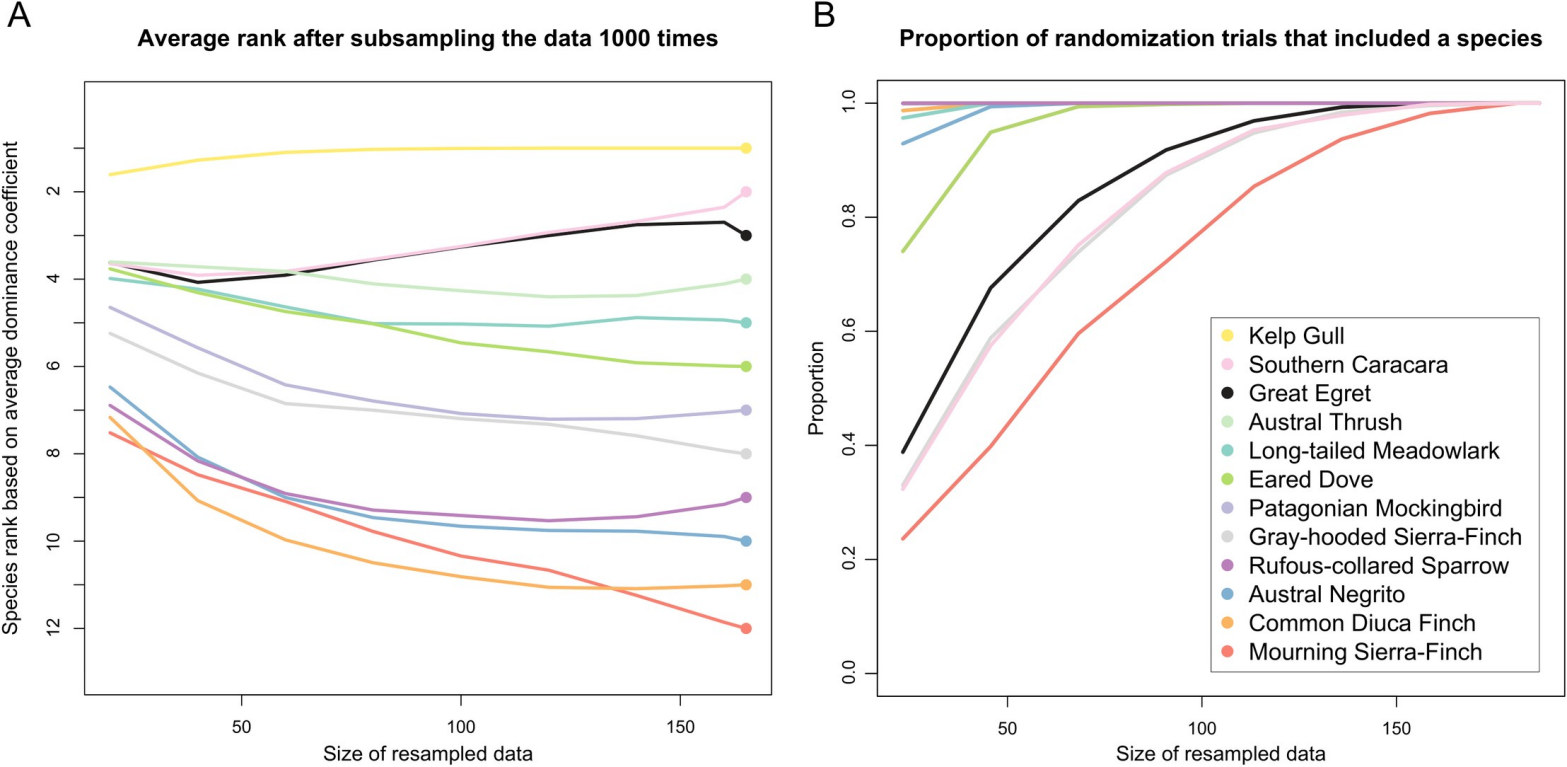
Robustness of the dominance hierarchy to variations in the number of observations. (A) Average species rank based on dominance coefficients calculated after subsampling the data 1000 times. The size of the subsampled datasets ranges from 20 observations to 160, in increments of 20. The dominance coefficients calculated without subsampling the data are shown in circles on the right. Species are color-coded as indicated in the legend. (B) The proportion of randomization trials that included each species as a function of the size of the subsampled datasets.

**Fig 2:**
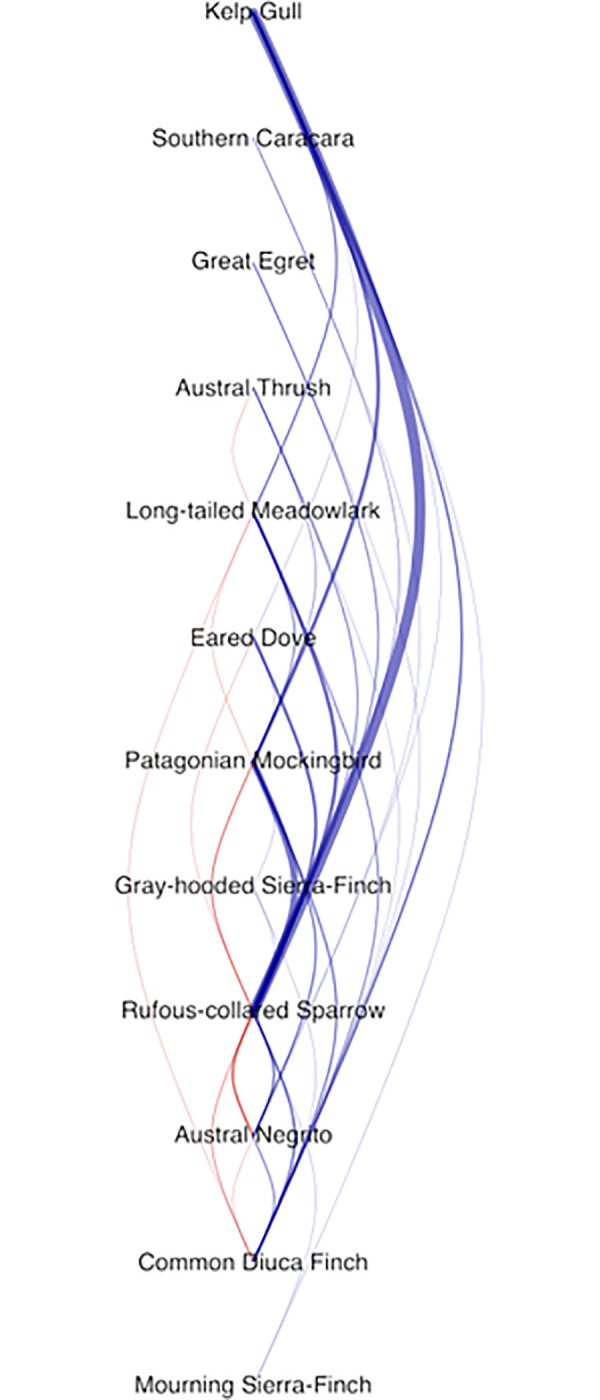
The overall dominance network organized by most dominant (top) to least dominant species (bottom). Blue lines on the right indicate displacement interactions where a more dominant bird displaced or rejected an attempted displacement by a subordinate bird. Red lines on the left indicate displacement interactions where a subordinate bird displaced or rejected an attempted displacement by a dominant bird. The thickness of the lines is proportional to the number of observed interactions between species.

We observed displacements involving 12 species; the kelp gull was the most dominant species and the mourning sierra-finch was the least dominant species recorded in the ranking (Table 1). We ranked the 12 species and found their rank in the hierarchy was relatively robust to variations in the amount of input data (Fig 1A), with very little variation in the relative ranking order after more than 100 observations were employed in the calculation. Although some species were represented by larger numbers of interactions than others in our dataset, the distribution of observations across taxa was such that the majority of species were present in our randomization trials once the number of observations was above 100 (Fig 1B). Using the ranking provided by the Bradley-Terry model, we constructed an attribute-ordered dominance network [25], displaying the 131 interspecific displacement interactions (Fig 2). The network is a directed acyclic graph, which means that the species follow a “pecking-order” hierarchy and that there are no cyclical “rock-paper-scissors” relationships [5, 24]. This visualization of the dominance hierarchy shows how the kelp gull was able to displace a large number of species. There were a small number of instances where a species that is lower in the rank displaced one with a higher dominance coefficient (Fig 2).

**Table 1:** Calculated ranking of 12 species based on displacements observed and dominance coefficients generated by the modified Bradley-Terry model.

Dominance Rank	Species	Latin Name	Dominance Coefficient	Average Body Mass (g)	Recorded Interactions (Wins and Losses)	Most Common Displacement Type (Losses)	Most Common Displacement Type (Wins)
1	Kelp gull	*Larus dominicanus*	20.99	957.0	42	N/A	Indirect
2	Southern crested caracara	*Caracara plancus*	18.984	1348.0	3	N/A	Indirect
3	Great egret	*Ardea alba*	18.97	873.5	4	N/A	Indirect
4	Austral thrush	*Turdus falcklandii*	1.371	93.9	7	Indirect	Indirect
5	Long-tailed meadowlark	*Leistes loyca*	1.169	113.0	33	Partial	Direct
6	Eared dove	*Zenaida auriculata*	0.66	136.0	10	Direct/indirect	Direct/indirect
7	Patagonian mockingbird	*Mimus patagonicus*	-0.66	57.8	51	Indirect	Indirect
8	Gray-hooded sierra-finch	*Phrygilus gayi*	-1.24	25.6	3	Indirect	Direct
9	Rufous-collared sparrow	*Zonotrichia capensis*	-2.41	21.1	118	Indirect	Direct
10	Austral negrito	*Lessonia rufa*	-2.50	13.4	20	Partial	Direct
11	Common diuca-finch	*Diuca diuca*	-3.22	36.8	37	Indirect	Partial
12	Mourning sierra-finch	*Rhopospina fruticeti*	-21.57	38.8	2	Direct/indirect	N/A

The table also shows the average mass for each species, the number of interactions that we observed involving each taxon (with two taxa being involved in each of the 131 observed interactions), and the most common type of displacement for both wins and losses.

**Fig 3:**
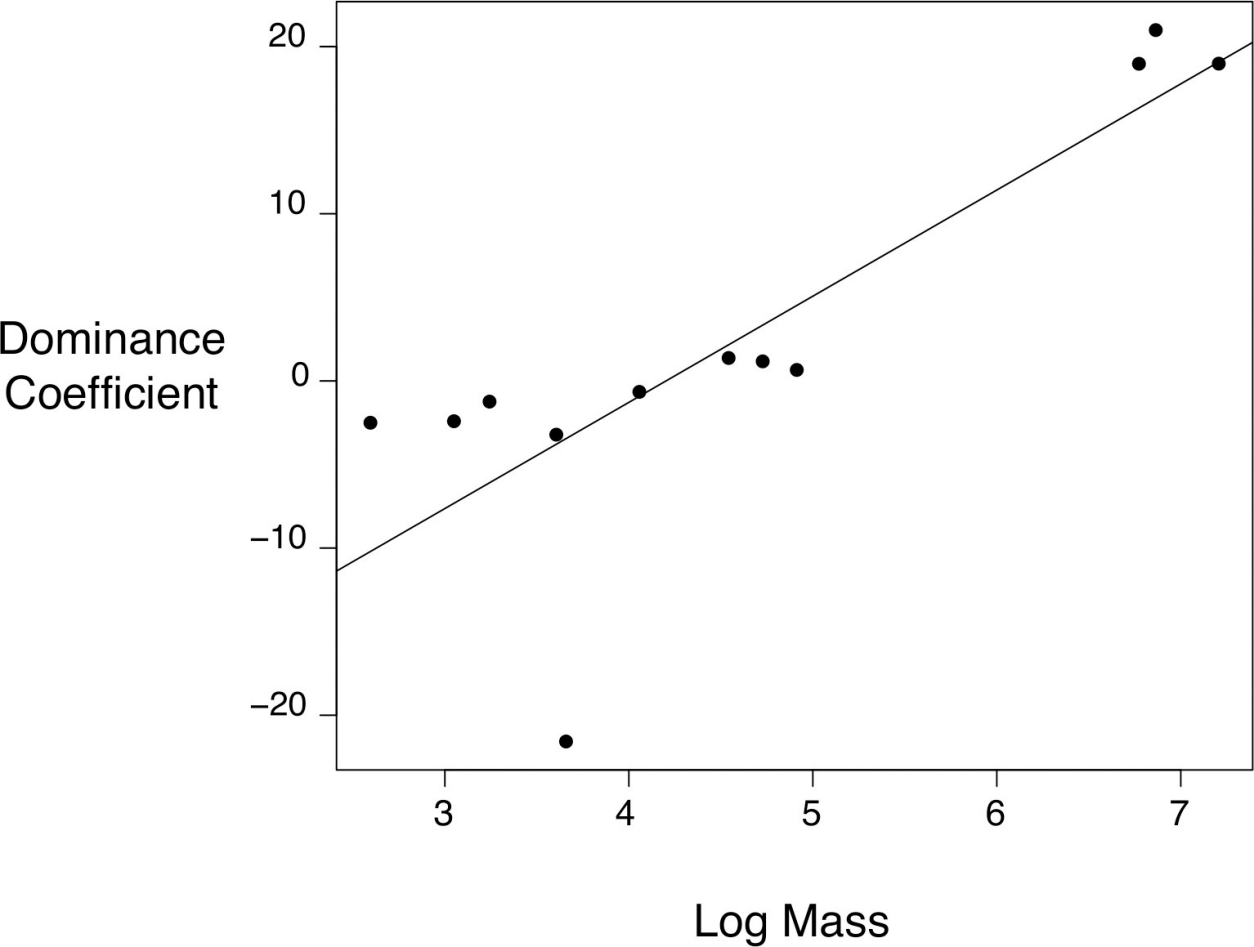
Recorded average mass of each species compared to calculated dominance coefficients. The outlier in the bottom of the plot corresponds to the Mourning Sierra-Finch.

We then explored the relationship between the position in the dominance hierarchy and body size and selected the PGLS regression considering an Ornstein-Uhlenbeck model of evolution (AIC value: 86.31vs 98.78 for Brownian Motion). According to this model, position in the dominance hierarchy is positively correlated with body mass (β = 6.35, *P* = 0.007, Fig 3)

**Fig 4:**
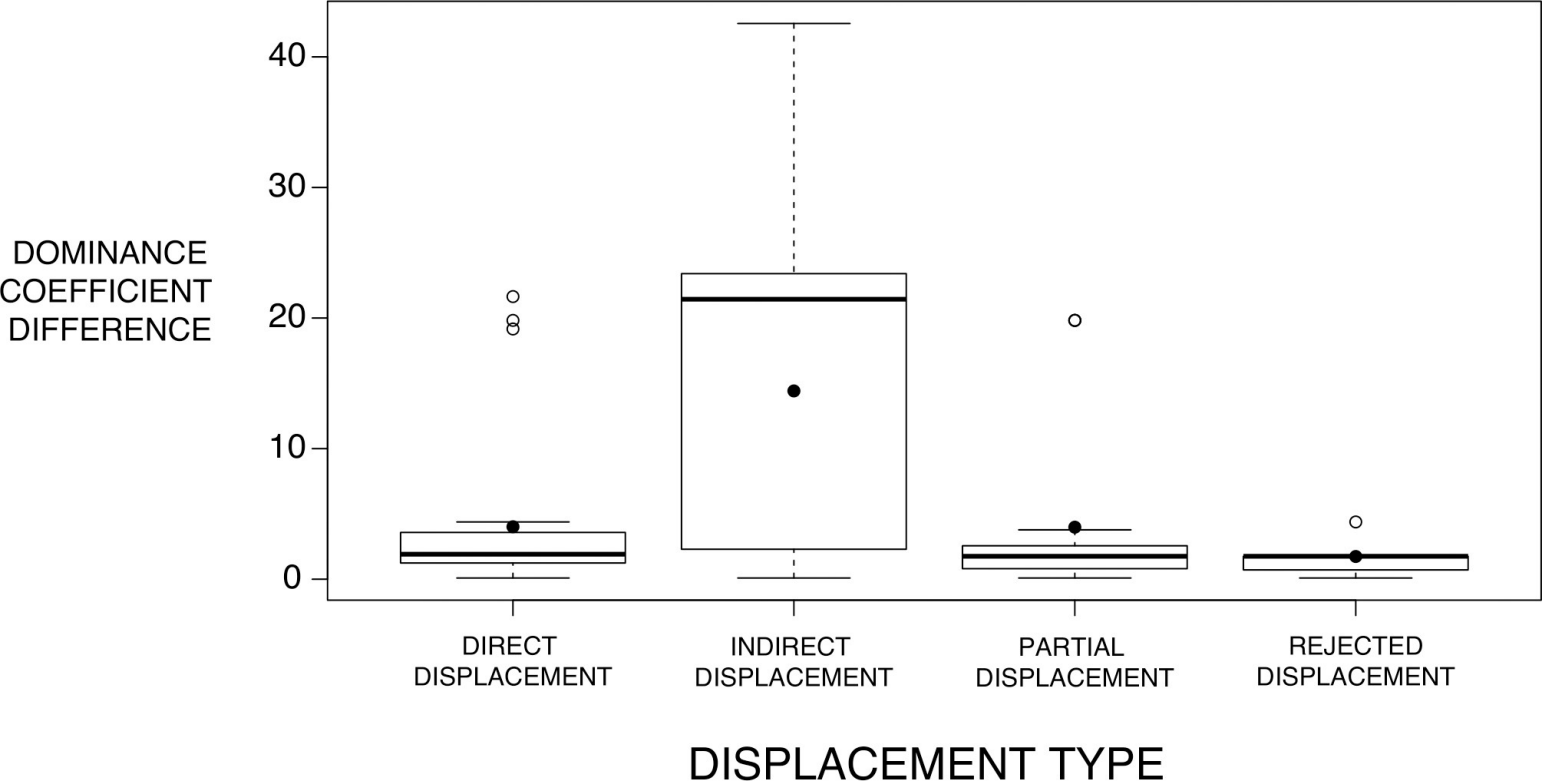
Difference in dominance between the two species involved in a given displacement interaction for each type of displacement. Horizontal lines indicate the median difference in dominance and the black circles represent the mean. The edges of the boxes are the 25 and 75 percentiles, the extremes of the distributions are denoted by the shorter horizontal lines, and white circles are outliers. The sample sizes for each category are (from left to right) 28, 76, 22, and 5.

The type of observed displacement was used as a predictor for the differences in dominance coefficients between the two interacting individuals. The results of the ANOVA showed that birds involved in indirect displacements had significantly larger differences in dominance coefficients compared to the other displacement types (F = 14.59, d.f. = 3, *p*<0.001) (Fig 4).

## Discussion

After recording displacements at a supplemental water source in an arid region, we constructed a dominance hierarchy comparable to the interspecific hierarchies commonly observed at food sources. Similar to other studies [5, 8, 12], we found that mass is a predictor of a species’ position in a dominance hierarchy (Fig 3). Our hierarchy is mostly linear with only a few displacements in which a subordinate species wins against a dominant species (Fig 2). Within this hierarchy, we found that kelp gulls are the most dominant species at this freshwater source. In addition, we found that indirect displacements occur more often between birds that have a large difference in dominance, indicating that displacement type can provide additional information when determining dominance hierarchies.

### Extremes of the hierarchy: Dominance of the kelp gull and subordination of the mourning sierra-finch

Our study shows that the kelp gull also dominates terrestrial birds when using a water source, limiting the access small terrestrial species have to it. This is particularly impactful because this water tank is the only abundant supplemental source of freshwater for several kilometers, so subordinate species must continue to use it, despite the risk posed by kelp gulls, or expend considerable effort to reach a safer source (where the ubiquitous kelp gulls will also be present). Because the subordinate species observed in this area are small terrestrial birds, they are at a higher risk of dehydration and require increased access to freshwater [15]. Subordinate birds are potentially experiencing dehydration more often due to the kelp gull’s ability to completely block or delay access to freshwater sources. In addition, the kelp gull’s dominance at this water source suggests that this species likely outcompetes other terrestrial birds for other scarce resources. Because kelp gulls are highly adaptable generalists that benefit from a variety of human waste sites [32–34], their population and range is expanding across coastal Patagonia [34]. Kelp gulls negatively impact coastal birds through predation and kleptoparasitism [33] and marine mammals through harassment and feeding on their skin and blubber [33, 35]. As kelp gull populations continue to grow through food supplementation derived from artificial food sources, they will likely affect subordinate terrestrial birds by outcompeting them for access to shared resources like freshwater.

It is unclear why the mourning sierra-finch had such a low dominance coefficient in comparison to the other species recorded, particularly the closely-related gray-hooded sierra-finch [36]. The disparity between the mourning sierra-finch and all other observed species could be due to limited data. There were very few displacement interactions recorded for this species (*N* = 2), both of which resulted in it being displaced. This species was only seen a few times in the area during the observation period, so the lack of data is likely due to a low number of mourning sierra-finches in this area. Further study in different areas and time periods is needed to confirm this pattern.

### Indirect displacements as an indicator of dominance

Generating dominance hierarchies that take into consideration different displacement types, such as those we defined in this study, will allow for a more in-depth understanding of interspecific interactions. For example, we observed that birds involved in indirect displacements had a significantly larger difference in dominance than in other displacement types. This is likely because highly dominant birds, like the kelp gull, are quickly recognized as dominant by subordinate species without the need for a direct aggressive interaction. Therefore, subordinate species are likely to preemptively leave before being subjected to aggressive behavior or predation. Most importantly, the dominance of the kelp gull was determined primarily through indirect displacements. Had we only used direct displacements to construct our hierarchy, like most other interspecific displacement-based studies have done [5, 12], much of the data supporting the most dominant species in our hierarchy would have been lost.

Additionally, indirect displacements are similar to active avoidance of predators, in which a prey animal detects and recognizes the danger a predator poses [19, 20]. In our study, the decision subordinate species made to leave preemptively when kelp gulls or other highly dominant species approached is very similar to foraging prey animals perceiving a threat and making decisions to lower the risk of predation [20], at the expense of the energetic cost of leaving the food source. The outcome of this decision varies depending on the energetic state of the prey animal; starving individuals are more likely to accept the high risk of predation in favor of foraging [20]. The outcome of the decision subordinate species make at water sources when dominant species arrive likely has similar consequences. Subordinate species must balance the cost of potential dehydration with risk of injury due to aggressive behavior from dominant species and should be more likely to stay at the water source despite the risks if already suffering from the effects of water imbalance.

### Future prospects

Our work found that dominance hierarchies can be observed at a supplemental water source in an arid region, and that behavioral responses depend on the relative position of both species in the dominance hierarchy. Future work is needed to expand the hierarchy to a larger number of Patagonian species and localities as there are multiple species inhabiting the study area that were not seen at the water tank during our observation period. In addition, comparing our results to hierarchies created in different time periods and areas would allow us to assess the stability of this hierarchy across different habitats and seasons. While there is some information on the stability of intraspecific hierarchies [9, 37, 38], more work needs to be done to understand the stability of interspecific relationships. Along the same lines, comparing our dominance hierarchy to one observed in the same area around a food source could determine if and how interspecific social dominance varies with different resources. It also remains to be determined if dominance hierarchies can be observed at water sources in environments where water is usually abundant.

## Supporting information

S1 Table Displacement data collected in Patagonia. (XLSX)
